# Comparative clinical study of the modified Broström procedure for the treatment of the anterior talofibular ligament injury—outcomes of the open technique compared to the arthroscopic procedure

**DOI:** 10.1007/s00264-023-05963-y

**Published:** 2023-09-05

**Authors:** LiLi Yang, QingFu Wang, YuanLi Wang, XiaoFang Ding, Huan Liang

**Affiliations:** 1 Department of Orthopedics, Beijing LongFu Hospital, Beijing, 100010 China; 2 Tendon Department of Traumatology The Third Affiliated Hospital of Beijing University of Traditional Chinese Medicine, Beijing, China

**Keywords:** Comparative clinical study, The modified Broström procedure, Anterior talofibular ligament injury, Outcomes of the open technique, The arthroscopic procedure

## Abstract

**Purpose:**

To observe the clinical efficacy and safety of arthroscopic-modified Broström surgery for the treatment of anterior talofibular ligament injury.

**Methods:**

The clinical data of 51 cases with anterior talofibular ligament injury were retrospectively analyzed, in which 23 patients were treated by arthroscopic-modified Broström surgery (arthroscopic surgery group) and 28 patients were treated by open-modified Broström surgery (open surgery group). The time to surgery, hospital stay, visual analog scale (VAS) scores of ankle pain, American Orthopaedic Foot and Ankle Society (AOFAS) ankle and hindfoot scores, and incidence rate of complications were compared between the two groups.

**Results:**

(1) General results: compared with open surgery group, arthroscopic surgery group had shorter time to surgery and hospital stay ((33.8 ± 6.7) min, (42.1 ± 8.5) min, *t* = 1.468, *P* = 0.001; (2.2 ± 1.4) d, (5.8 ± 1.6) d, *t* = 1.975, *P* = 1.975, *P* = 0.002). (2) VAS scores of ankle pain: there was an interaction effect between the time and group factors (*F* = 0.378, *P* = 0.018); overall, there was no statistically significant difference in VAS scores of ankle pain between the two groups, i.e., there was no grouping effect (*F* = 1.865, *P* = 0.163); there was statistically significant difference in VAS score of ankle pain at different time points before and after operation, i.e., there was a time effect (*F* = 1.675, *P* = 0.000); the VAS scores of ankle pain showed a decreasing trend with time in both groups, but the decreasing trend was not completely consistent between the two groups ((7.78 ± 1.23), (1.23 ± 1.24), (1.03 ± 0.35), (1.01 ± 0.28), *F* = 0.568, *P* = 0.000. (7.45 ± 1.43), (1.45 ± 1.87), (1.23 ± 0.55), (1.04 ± 0.37), *F* = 1.358, *P* = 0.000); there was no statistically significant difference in VAS score of ankle joint pain between the two groups six and 12 months before and after surgery (*t* = 2.987, *P* = 0.055; *t* = 1.654, *P* = 2.542; *t* = 0.015, *P* = 0.078); the VAS scores of ankle pain in the arthroscopic surgery group was lower than that in the open surgery group three months after operation (*t* = 1.267, *P* = 0.023). (3) AOFAS ankle and hindfoot scores: there was an interaction effect between time and grouping factors (*F* = 2.693, *P* = 0.027); overall, there was no statistically significant difference in the AOFAS ankle and hindfoot scores between the two groups, i.e., there was no grouping effect (*F* = 1.983, *P* = 0.106); there was statistically significant difference in the AOFAS ankle and hindfoot scores at different time points before and after surgery, i.e., there was a time effect (*F* = 34.623, *P* = 0.000); the AOFAS ankle and hindfoot scores of the two groups showed an increasing trend with time, but the increasing trend of the two groups was not completely consistent ((48.19 ± 12.89), (89.20 ± 8.96), (90.24 ± 7.89), (91.34 ± 9.67), *F* = 25.623, *P* = 0.000; (49.35 ± 13.28), (86.78 ± 12.34), (88.78 ± 9.78),(91.43 ± 7.98), *F* = 33.275, *P* = 0.000); there was no statistically significant difference in the AOFAS ankle and hindfoot scores between the two groups 12 months before/after surgery (*t* = 2.145，*P* = 0.056；*t* = 2.879，*P* = 0.389); compared with open surgery group, the arthroscopic surgery group had higher AOFAS ankle and hindfoot scores 3/6 months after surgery (*t* = 1.346, *P* = 0.014; *t* = 1.874, *P* = 0.028).

**Conclusion:**

For the treatment of anterior talofibular ligament injury, arthroscopic surgery group is superior to open surgery group in ankle pain relief and functional recovery and has shorter operation time and hospital stay compared with open surgery group.

## Introduction

Ankle sprain can easily lead to the injury of the lateral ligament complex of the ankle, which may develop into chronic ankle instability if not treated in time. The lateral ligament of the ankle joint consists of three ligaments, namely the anterior talofibular ligament, posterior talofibular ligament, and calcaneus fibular ligament. The anterior talofibular ligament is the main component of the lateral ligament complex of the ankle joint and is also the most vulnerable ligament to ankle sprain. For confirmed anterior talofibular ligament injuries, surgical treatment should be used clinically [1].

The commonly used surgical methods include anatomical reconstruction and non-anatomical reconstruction of the anterior talofibular ligament. Patients after nonanatomical reconstructive surgery are prone to dorsiflexion-extensor dysfunction and ankle inversion complications, and, at present, clinical use is rare.

Broström surgery is an effective surgical method in anatomical reconstruction surgery, which involves suturing the anterior talofibular ligament to the distal end of the fibula through bone piercing and directly suturing the broken segment of the ligament to treat ankle instability. The improved Broström surgery is based on the Broström surgery by adding sutures to strengthen the extensor retinaculum. The open-modified Broström operation for the treatment of anterior talofibular ligament injury achieve good clinical efficacy, but there are also complications such as delayed wound healing and deep vein thrombosis [2–9]. With the rapid development of arthroscopic technology, the arthroscopic-modified Broström operation has gradually been widely used in clinical practice [10–12]. In order to explore a better method for the treatment of anterior talofibular ligament injury, the case data of patients with anterior talofibular ligament injury treated by arthroscopic-modified Broström operation and open-modified Broström operation was retrospectively analyzed, and the clinical efficacy and safety of the two methods were compared.

## Method

### General information

The patients with anterior talofibular ligament injury who were hospitalized in the hospital from January 2018 to January 2021 were eligible for the study. When calculating the sample size, the effective rates of the two surgical methods were used as the main efficacy indicator. Referring to relevant literature, the effective rates of the arthroscopic group and the open surgery group were 92% and 83%, respectively. Taking into the sample size calculation formula, 23 cases were required for the arthroscopic group and 28 cases were required for the open surgery group. The trial plan was reviewed and approved by the medical ethics committee of the hospital.

### Study type

This study is a retrospective cohort study.

### Inclusion criteria

(1) The diagnosis of anterior talofibular ligament injury was confirmed with MRI and arthroscopy; (2) patients with chronic ankle instability who do not show significant improvement after conservative treatme; (3) patient age ≥ 18 years old; (4) patients were under the treatment of arthroscopic-modified Broström operation or open-modified Broström operation; (5) follow-up duration >12 months; (6) the case data was complete.

### Exclusion criteria

(1) Patients with periankle fracture; (2) ankle joint deformity; (3) the case data had common sense or logic errors.

The patients treated with arthroscopic-modified Broström operation were included in the arthroscopic operation group, and the patients treated with open-modified Broström operation were included in the open operation group.

### Treatment

**Fig. 1:**
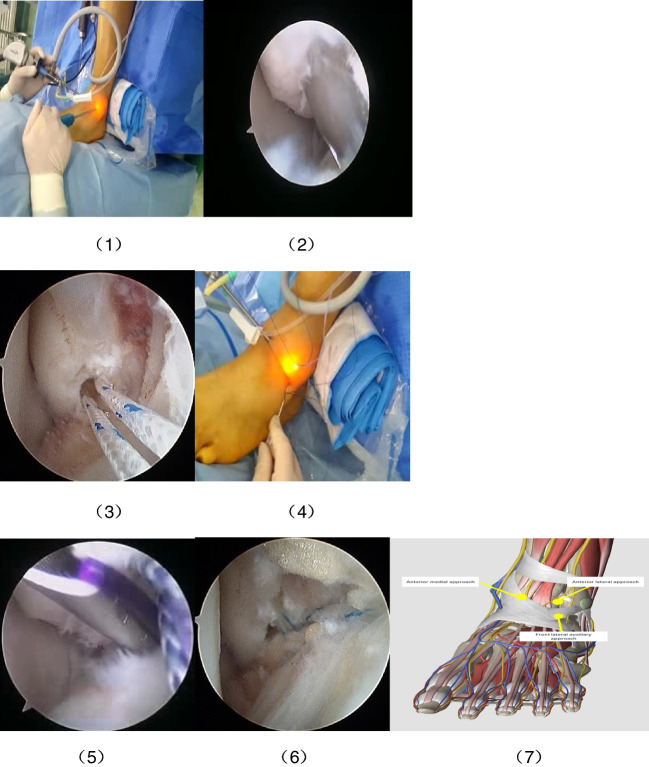
Intraoperative picture of the arthroscopic-modified Broström procedure. **1** Find the anterolateral and anterolateral approaches to the ankle joint. **2** Anchor guide was placed during the operation. **3** The anchor tail line was pulled out after inserting the suture anchor. **4** The anterior talofibular ligament was penetrated with 18G hollow needle. **5** During the operation, the residual end of the anterior talofibular ligament was penetrated by PDS II suture with a puncture needle. **6** Two anchor nail tail line knotted. **7** Anatomical structure and surgical approach

The arthroscopic-modified Broström operation adopted nerve block or intraspinal anaesthesia. The patient was placed in a supine position with a pneumatic tourniquet on the root of the affected thigh, which was routinely disinfected and covered with towels. The distal fibula, fibular tendon, and cutaneous nerve course around the ankle joint were marked on the body surface. The affected ankle was kept in the neutral position, the anteromedial and anterolateral arthroscopic approaches at the lateral tibialis anterior tendon, the level of the ankle joint line, and 1.0–1.5 cm anterior to the fibula end were established (Fig. 1 (1)). The anteromedial approach was used for surgical observation, and the anterolateral approach was used for surgical operation. The arthroscope with diameter of 2.7 mm and inclination angle of 30° was used. First, the quality of the residual end of the anterior talofibular ligament and the cartilage damage were evaluated under arthroscopy, inflammatory synovial tissue and osteophytes were removed. Under arthroscopy, an anchor guide was placed at the distal end of the fibula, an absorbable band anchor with a diameter of 2.9 mm was inserted along the anchor guide, and the tail line of the anchor was pulled out (Fig. 1 (1), (2)). A hollow needle with PDS II suture was passed percutaneously from approximately 1 cm anteroinferiorly to the end of the fibula (Fig. 1 (4)), and PDS II suture was penetrate through the residual end of the anterior talofibular ligament and part of the lower extensor retinaculum with a hollow needle (Fig. 1 (5)). PDS II suture was pulled out with grasper from the anterolateral approach, bound to the tail line of the first anchor, pulled from the other end of the PDS II suture, and the tail line of the anchor was passed from the residual end of the anterior peroneal ligament, and then penetrated the skin from the hollow needle entry point (Fig. 1 (6)). An incision about 2 mm long from the puncture point of the hollow needle was made, the subcutaneous tissue between the incision and the anterolateral approach was separated bluntly and the other end of the tail line of the first anchor was pulled from the skin. The hollow needle was insert again at about 5 mm above the first the hollow needle entry point, and the same steps were repeat to pass the tail line of the second anchor through the residual end of the anterior peroneal ligament and part of the lower extensor retinaculum. It was noted that the two anchor tail lines should be spaced a certain distance apart from the stitches on the anterior peroneal ligament. The ankle joint was kept in the neutral plantar flexion position and slightly everted, and the tail line of the two anchors was tied, respectively. The drawer test and varus stress test of the ankle joint were performed to ensure the stability of the ankle joint. Then, the drainage tube was placed, the incision was sutured, the elastic bandage was bandaged, and the ankle joint was fixed in the neutral position with a brace.

### Open-modified Broström operation

**Fig. 2:**
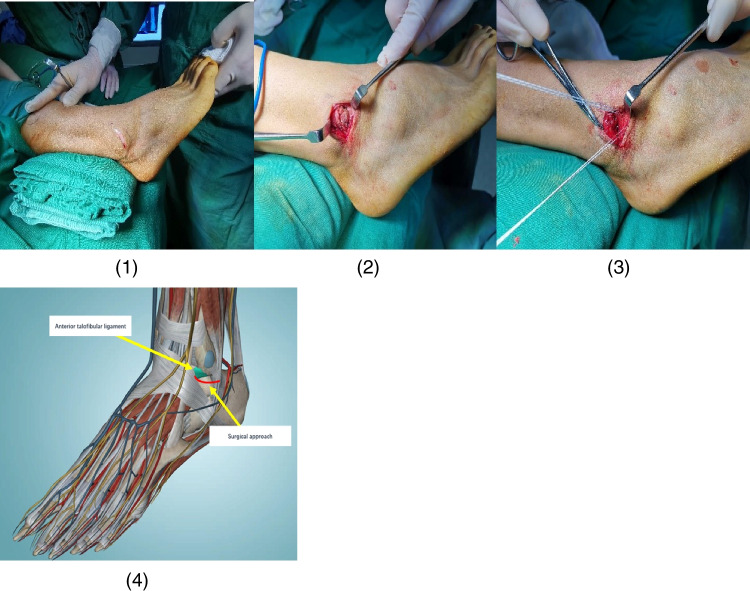
Intraoperative photograph of open anterior talofibular ligament repair. **1** An arc incision at the front edge of fibula was made. **2** The joint capsule was opened to expose the anterior talofibular ligament. **3** The anterior talofibular ligament was sutured after the anchor was inserted. **4** Anatomical structure and surgical approach

Local nerve block or intraspinal anaesthesia was adopted. The patient was placed in a supine position with a pneumatic tourniquet on the root of the affected thigh, which was routinely disinfected and covered with towels. An arc incision of about 5 cm in length was made in the anteroinferior aspect of the distal fibula on the affected side (Fig. 2 (1)), the skin, subcutaneous tissue and deep fascia were incised, and the joint capsule was opened to expose the anterior talofibular ligament (Fig. 2 (2)), paying attention to protecting the sural nerve and superficial peroneal nerve branches. The scar tissue in the joint capsule was cleaned to explore the injury degree of the anterior talofibular ligament and the surrounding joint capsule. The anchor was placed on the anterior side of the distal fibula, and the anterior talofibular ligament and part of the extensor retinaculum were tightened and sutured with the anchor tail line (Fig. 2 (3)). The drawer test and varus stress test of the front ankle joint were performed. After confirming good stability of the ankle joint, a drainage tube was placed, the incision was sutured layer by layer, covered with sterile dressing, wrapped with elastic bandage, and the ankle joint was fixed in a neutral position with a brace.

### Postoperative treatment

The patients in the two groups raised the affected limb after the operation, maintained the affected limb in the neutral position with ankle brace, and antibiotics and anti-swelling drugs were administrated routinely; the drainage tube was removed within 48 h after operation; the isometric contraction training of lower limb muscles was performed on the second day after operation; the ankle brace was worn for partial weight bearing two weeks after the operation, and rehabilitation training was conducted under the guidance of the rehabilitation instructor; endurance and balance training were carried out four weeks after operation, and full weight bearing was performed gradually; adaptive activities such as jogging were performed 12 weeks after operation.

### Primary and secondary outcomes

The duration of surgery, hospital stay, VAS scores of ankle pain, AOFAS ankle and hindfoot scores [13], and the incidence rate of complications were compared between the two groups. Perform efficacy evaluation 3 months after surgery.

### Statistical methods

SPSS27.0 statistical software was used for analyses. χ^2^ test was used to compare the gender and injury side between the two groups; *t*-test was used to compare age and body mass index between the two groups; analysis of variance (ANOVA) was used repeatedly to compare VAS scores of ankle pain and AOFAS ankle and hindfoot scores. The test level was *α* = 0.05.

## Results

A total of 51 patients were included, 23 in the arthroscopic surgery group and 28 in the open surgery group. The differences of baseline information between the two groups were not statistically significant and were comparable (Table 1).

**Table 1:** Baseline information of patients with anterior talofibular ligament injury in 2 groups

Group	Sample size/example	Age ($\overline{x}\pm s$, years)	Gender/example		Body mass index ($\overline{x}\pm s$, kg·m^−2^)	Injury side/case	
			Male	Female		Left side	Right side
Arthroscopic surgery group	23	46.7±16.7	11	12	28.1±6.38	10	13
Open surgery group	28	48.9±17.3	16	12	29.4±5.76	14	14
Test statistic		*t*=1.267	*χ*^2^=0.440		*t* = 2.538	*χ*^2^ = 1.125	
*P*-value		0.280	0.507		0.807	0.762	

### General results

Compared with open surgery group, arthroscopic surgery group had shorter time to surgery and hospital stay ((33.8 ± 6.7) min, (42.1 ± 8.5) min, *t* = 1.468, *P* = 0.001; (2.2 ± 1.4) d, (5.8 ± 1.6) d, *t* = 1.975, *P* = 0.002).

### Results of efficacy and safety evaluation

#### Ankle pain VAS score

There was no statistically significant difference in VAS scores of ankle joint pain between the two groups of patients; There was a statistically significant difference in VAS scores of ankle joint pain at different time points before and after surgery; The VAS score of ankle pain in both groups of patients showed a downward trend over time, but the downward trend was not completely consistent in both groups; There was no statistically significant difference in VAS scores of ankle joint pain between the two groups of patients before surgery, six months after surgery, and 12 months after surgery; three months after surgery, the VAS score of ankle pain in the arthroscopic surgery group was lower than that in the open surgery group (Table 2).

**Table 2:** Visual analog scale scores of ankle pain before and after surgery in 2 groups of patients with anterior talofibular ligament injury

Group	Sample size/example	Visual analog scale score for ankle pain ($\overline{x}\pm s$, points)					*F*-value	*P*-value
		Preoperative	3 months after surgery	6 months after surgery	12 months after surgery	Summation		
Arthroscopic surgery group	23	7.78±1.23	1.23±1.24	1.03±0.35	1.01±0.28	2.75±0.70	0.568	0.000
Open surgery group	28	7.45±1.43	1.45±1.87	1.23±0.55	1.04±0.37	2.81±0.99	1.358	0.000
Summation	51	7.65±1.87	1.38±1.36	1.14±0.46	1.03±0.31	2.78±0.84	1.675^a^	0.000^a^
Test statistic		*t* = 2.987	*t* = 1.267	*t* = 1.654	*t* = 0.015	1.865^a^	*F* = 0.378^b^， *P* = 0.018^b^	
*P*-value		0.055	0.023	2.542	0.078	0.163^a^		

^a^*F-* and *P*-values for main effects

^b^*F*- and *P*-values for interaction effects

#### AOFAS ankle and hindfoot scores

The overall comparison of AOFAS ankle and hindfoot scores between the two groups of patients showed no statistically significant differences between the groups. The differences in AOFAS ankle and hindfoot scores at different time points before and after surgery were statistically significant; The AOFAS ankle and hindfoot scores of both groups of patients showed an upward trend over time, but the upward trend was not completely consistent between the two groups; There was no statistically significant difference in AOFAS ankle and hindfoot scores between the two groups of patients 12 months before and after surgery; at three months and six months after surgery, the AOFAS ankle and hindfoot scores of patients in the arthroscopic surgery group were higher than those in the open surgery group (Table 3).

**Table 3:** American Foot and Ankle Society ankle and hindfoot scores before and after surgery in patients with anterior talofibular ligament injuries in 2 groups

Group	Sample size/example	American Foot and Ankle Society ankle and hindfoot score ($\overline{x}\pm s$, points)					*F*-value	*P*-value
		Preoperative	3 months after surgery	6 months after surgery	12 months after surgery	Summation		
Arthroscopic surgery group	23	48.19±12.89	89.20±8.96	90.24±7.89	91.34±9.67	80.38±9.79	25.623	0.000
Open surgery group	28	49.35±13.28	86.78±12.34	88.78±9.78	91.43±7.98	79.16±11.86	33.275	0.000
Summation	51	49.78±19.47	87.78±24.62	89.78±15.38	92.72±21.79	79.81±10.11	34.623^a^	0.000^a^
Test statistic		*t* = 2.145	*t* = 1.346	*t* = 1.874	*t* = 2.879	1.983^a^	*F* = 2.693^b^， *P* = 0.027^b^	
*P*-value		0.056	0.014	0.028	0.389	0.106^a^		

^a^*F*- and *P*-values for main effects

^b^*F*- and *P*-values for interaction effects

### Complication rate

In the arthroscopic surgery group, 1 patient developed superficial peroneal nerve irritation symptoms, which disappeared after treatment with trophic nerve drugs; one patient developed chronic local pain syndrome, which disappeared after oral NSAIDs and physiotherapy. In the open surgery group, there was one case of delayed healing of the incision, which healed after enhanced dressing change; one case of deep venous thrombosis, which disappeared after anticoagulant thrombolytic therapy; one case of chronic local pain syndrome, which disappeared after oral NSAIDs and physiotherapy. There was no significant difference in the incidence rate of complications between the two groups (χ^2^ = 2.526, *P* = 0.112).

## Discussions

Ankle sprains are the most common condition in foot and ankle surgery, occurring in approximately 30,000 people per day in the USA. Due to differences in the medial and lateral anatomy of the ankle joint, 85% of ankle sprains are lateral ligament injuries. The anterior talofibular ligament, the calcaneal ligament, and the posterior talofibular ligament constitute the anterior, middle, and posterior bundles of the lateral ligaments of the ankle, respectively, of which the anterior talofibular ligament is the weakest and most easily damaged. Anterior talofibular ligament injuries account for about two-thirds of lateral ankle ligament injuries. Most patients with ankle sprains can obtain good clinical outcomes after non-surgical treatment, but 10 to 30% of patients still have recurrent sprains after treatment with clinical manifestations such as pain, swelling, and fear of walking on the lateral side of the ankle. If the clinical manifestation lasts for more than six months, it is called chronic lateral ankle instability.

Injuries to the lateral ankle ligament complex may develop into chronic ankle joint instability [14]. Stress imaging is often used clinically to evaluate injury to the lateral ankle ligament complex: the ankle joint is varus to the greatest extent, and an anteroposterior radiograph of the ankle joint is taken to measure the talus varus angle [15]. The talus varus angle on the affected side is 8 to 10° greater than that on the unaffected side suggesting lateral collateral ligament injury, 15° greater than that on the unaffected side suggesting anterior talofibular ligament injury, 15 to 30° greater than that on the unaffected side suggesting calcaneofibular ligament and anterior talofibular ligament injury, and 30° greater than that on the unaffected side suggesting lateral ankle ligament injury [15]. In addition, a displacement of the talus of more than 3 mm in the anterior drawer test of the ankle also indicates an injury to the anterior talofibular ligament.

Ankle sprains occur repeatedly on a daily basis, which easily leads to damage to the medial and lateral stable structures of the ankle joint. The lateral collateral ligament has been reported to account for 90% of ankle ligament injuries and 10–30% of patients with acute injuries develop chronic ankle instability. The anterior talofibular ligament is the weakest bundle of the lateral collateral ligaments of the ankle joint. Its general shape showed a flat quadrilateral with wide ends and a slightly narrower midsection. It is primarily responsible for limiting talus advancement and preventing excessive varus of the foot.

Although anterior talofibular ligament injuries are common in orthopaedic clinics, some patients develop various sequelae at the late stage of injury due to insufficient attention and improper early management. Therefore, understanding the clinical significance of the anterior talofibular ligament, early diagnosis, and early treatment at the early stage of injury can effectively prevent and treatment the occurrence of sequelae. Patients complained of a history of plantar flexion and varus sprain of the ankle joint, accompanied by swelling, pain, and tenderness in the anterior part of the lateral malleolus, and stress radiography of sagittal plane should be performed Anterior talofibular ligament injury should be considered when the talus is displaced anteriorly more than 3 mm, and ankle MRI and ankle arthrography can assist in the diagnosis. For new ankle sprains, depending on the extent of the anterior talofibular ligament injury, different methods of treatment are generally used in China, such as taping, plaster fixation in functional position and surgical suturing with plaster fixation. For the conservative treatment, some patients may have ankle instability, swelling after activity, pain, ankle stiffness, and traumatic arthritis, while surgical treatment can achieve good stability with a longer recovery period. Patients with habitual ankle instability are often accompanied by intraarticular synovitis, osteophyte formation, talar cartilage injury and other lesions in addition to ligament injury. Arthroscopic exploration can be performed for hyperplastic synovial shaving, osteophyte resection, loose body removal, injured chondroplasty, and other joint debridement, which is conducive to the relief of symptoms. At the same time, it is difficult to make a definite diagnosis of lateral ankle ligament injury by clinical signs and imaging. Arthroscopically, however, it can clearly evaluate the degree of joint capsule and ligament damage to further confirm the diagnosis. For old ankle sprains, surgical treatment is not always necessary for patients with complete ligament rupture. A significant number of patients present with incomplete fractures by varus stress radiographs as well as anterior drawer tests. However, laxity of the anterior talofibular ligament, ankle instability, and pain develop later. For these patients, active surgical treatment should be adopted, and the short-term surgical efficacy is better.

At present, there are many surgical procedures for chronic ankle instability, and these procedures and their modified surgical procedures can be divided into three categories, namely anatomic repair, non-anatomic reconstruction, and anatomic reconstruction [16]. Anatomical repair refers to the in situ repair of the injured ligament to restore the normal anatomical structure and mechanical relationship, including shortening the suture, strengthening the suture with adjacent tissue, or fixing the ligament to the bone surface. Broström-Gould procedure is one of the classic procedures, which can strengthen the injured anterior talofibular ligament through the inferior extensor retinaculum and enhance the anti-varus strength of the ligament without sacrificing other normal structures, with little injury and simple operation. Russo’s long-term follow-up of 18 athletes with chronic ankle instability who underwent this procedure showed an excellent rate of 94.5% [17]. Anatomic reconstruction should be considered when the patient is in the condition the poor quality of the ligament stump, excessive obesity, systemic ligament laxity, and primary surgical failure [18]. Anatomic reconstruction is performed by implanting autologous or allogeneic tendons in situ into the starting and ending points of the injured ligament to restore the anatomical and mechanical relationship between the anterior talofibular ligament and the calcaneofibular ligament.

The efficacy of open surgery for the treatment of old avulsion fractures of the external ankle complicated by ankle instability has been preliminarily verified, while arthroscopic surgery for lesions caused by chronic ankle instability has more advantages, with the improvement of arthroscopic instrumentation, arthroscopic surgery leaves no skin scars, does not affect the appearance, and has a relatively rapid postoperative recovery while obtaining the expected clinical efficacy. At present, there are many arthroscopic surgical methods for avulsion fractures of the lateral malleolus caused by anterior talofibular ligament injury. Broström procedure, first reported in 1966, allows a direct repair of the anterior talofibular ligament; however, it is more difficult to repair the anterior talofibular ligament directly by this procedure, and the strength of the ligament is reduced after surgery. Broström-Gould procedure is based on the Broström procedure with an enhanced suture of the extensor retinaculum with the peroneal periosteum, which is currently considered the gold standard for repairing of the lateral ankle ligament. With the development of ankle arthroscopy, total ankle arthroscopic procedure for the treatment of anterior talofibular ligament injuries and chronic instability of the ankle joint has become a hot topic in this discipline. With the total ankle arthroscopic surgery, the treatment of minimal change disease is more meticulous with clearer surgical field, smaller surgical trauma, lower incidence of vascular and nerve injury, and no skin scars will be left after surgery.

The modified Broström procedure is the most commonly used method for anatomic reconstruction of the anterior talofibular ligament, which is mainly divided into two main parts: first, the ligament is sutured to the inferior edge of the fibula with non-absorbable sutures; second, the lateral part of the inferior extensor muscle support band is separated, sutured to the edge of the fibula and covered on the anterior talofibular ligament to increase the strength of the ligament [14, 15]. Compared with non-anatomic reconstructive surgery, the modified Broström procedure does not require the use of the peroneal tendon, which avoids issues such as medical injury due to the use of the peroneal tendon, and the anatomic reconstruction does not restrict the movement of the subtalar joint and has no effect on the mobility of the ankle joint. With the continuous development of arthroscopic techniques, arthroscopic-modified Broström procedure has been gradually applied clinically to treat anterior talofibular ligament injuries. The arthroscopic modification of the Broström procedure is gradually being used clinically to treat anterior talofibular ligament injuries. Yi Gang et al. [19] retrospectively analyzed 65 patients with anterior talofibular ligament injuries treated with the modified Broström surgery, of which 35 patients were treated with arthroscopic-modified Broström surgery, 30 patients were treated with open-modified Broström surgery. The results showed that the AOFAS ankle and hindfoot scores and Karlsson ankle function scores in the arthroscopic surgery group were higher than in the conventional surgery group at two weeks after surgery. At three months after surgery and the final follow-up, there were no significant differences in AOFAS ankle and hindfoot scores and Karlsson ankle function scores between the two groups. Moorthy et al. [20] systematically evaluated the clinical efficacy and safety of modified Broström surgery and open Broström surgery for anterior talofibular ligament injury, and their study showed that the long-term clinical efficacy of the two methods were comparable, but the complication rate of arthroscopic-modified Broström surgery was lower than that of open-modified Broström surgery. The results of this study showed compared with open surgery group, the arthroscopic surgery group had lower VAS score of ankle joint pain three months after surgery; compared with open surgery group, the arthroscopic surgery group had higher AOFAS ankle and hindfoot scores 3/6 months after surgery; suggesting that ankle pain relief and functional recovery were better in arthroscopic surgery group at the early postoperative period, which was related to the smaller trauma of arthroscopic surgery.

## Conclusion

This study shows that compared with open-modified Broström procedure, arthroscopic-modified Broström procedure has shorter time to surgery and hospital stay. Besides, regarding ankle pain relief and functional recovery, open-modified Broström procedure is more effective than the open-modified Broström procedure for the treatment of anterior talofibular ligament injuries. However, the sample size of this study is small, and the conclusions need to be further validated with a higher-quality multicenter, large-sample randomized controlled trial.
